# miRNATissueAtlas 2025: an update to the uniformly processed and annotated human and mouse non-coding RNA tissue atlas

**DOI:** 10.1093/nar/gkae1036

**Published:** 2024-11-14

**Authors:** Shusruto Rishik, Pascal Hirsch, Friederike Grandke, Tobias Fehlmann, Andreas Keller

**Affiliations:** Clinical Bioinformatics, Center for Bioinformatics, Saarland University, 66123 Saarbrücken, Germany; Clinical Bioinformatics, Center for Bioinformatics, Saarland University, 66123 Saarbrücken, Germany; Clinical Bioinformatics, Center for Bioinformatics, Saarland University, 66123 Saarbrücken, Germany; Retro Biosciences, Redwood City, CA, 94063, United States; Clinical Bioinformatics, Center for Bioinformatics, Saarland University, 66123 Saarbrücken, Germany; Department of Neurology and Neurobiology, Stanford University, Stanford, CA, 94305, USA

## Abstract

MiRNAs represent a non-coding RNA class that regulate gene expression and pathways. While miRNAs are evolutionary conserved most data stems from *Homo sapiens* and *Mus musculus*. As miRNA expression is highly tissue specific, we developed miRNATissueAtlas to comprehensively explore this landscape in *H. sapiens*. We expanded the *H. sapiens* tissue repertoire and included *M. musculus*. In past years, the number of public miRNA expression datasets has grown substantially. Our previous releases of the miRNATissueAtlas represent a great framework for a uniformly pre-processed and label-harmonized resource containing information on these datasets. We incorporate the respective data in the newest release, miRNATissueAtlas 2025, which contains expressions from 9 classes of ncRNA from 799 billion reads across 61 593 samples for *H. sapiens* and *M. musculus*. The number of organs and tissues has increased from 28 and 54 to 74 and 373, respectively. This number includes physiological tissues, cell lines and extracellular vesicles. New tissue specificity index calculations build atop the knowledge of previous iterations. Calculations from cell lines enable comparison with physiological tissues, providing a valuable resource for translational research. Finally, between *H. sapiens* and *M. musculus*, 35 organs overlap, allowing cross-species comparisons. The updated miRNATissueAtlas 2025 is available at https://www.ccb.uni-saarland.de/tissueatlas2025.

## Graphical Abstract

**Graphical Abstract:**
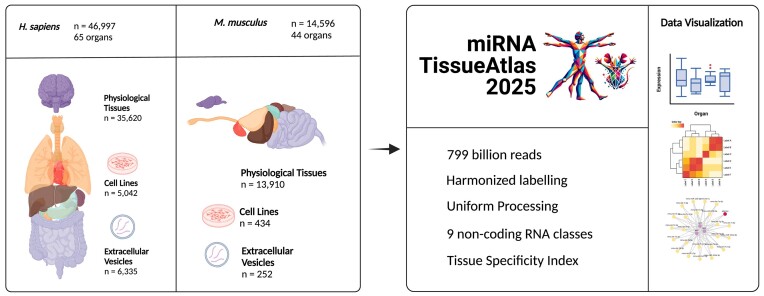

## Introduction

Non-coding RNAs such as miRNAs are critical for the modulation of protein expression levels in tissues (1,2). MiRNAs represent one of the best studied classes across non-coding RNAs, differing from other small non-coding RNA in terms of their size, biogenesis, targeting mechanism and downstream function. They are 21–23 nt short sequences that target the 3′ UTRs of mammalian mRNA, bind to them and degrade them or disrupt their translation (3). Families of miRNA can have over 400 such targeting interactions, with the interactions being evolutionarily conserved (4). These interactions can result in up or downregulation of entire pathways, making them relevant to the study of diseases, either as drug targets or biomarkers (5,6). MiRNA sequences themselves are also evolutionarily conserved. Two thirds of all known miRNAs are similar between large primates such as *Homo sapiens* and *Mus musculus* (7,8). MiRNAs that are diverging between species can take on species-specific functionality. For example, recently evolved *H. sapiens* miRNAs were shown to be enriched for neuronal functions (7), hinting a possible role of miRNA in the intelligence explosion in humans. Sequence variation in a miRNA, even at a single base pair, can generate isomiRs (9) which can alter its downstream targeting, e.g. by seed shifting, and give rise to new functionality. If these functionalities prove adaptive, these isomiRs can undergo natural selection, become fixed in a species, and give rise to new miRNAs over time (10). The biogenesis of isomiRs can happen under other conditions as well and are also relevant to the study of diseases, being especially well-studied in cancer (11). MiRNAs are exclusively transcribed from the nuclear genome but are localized to both cytoplasmic and mitochondrial subcellular compartments (12). This contrasts with other non-coding RNAs such as rRNAs and tRNAs, which have copies both on the nuclear and mitochondrial genomes (13,14). The processing of tRNA can also generate tRFs in both the cytoplasm and mitochondria and represents an emerging class of non-coding RNA previously confused for miRNA (15,16). Moreover, the near ubiquitious presence of miRNA offer a practical tool for modulating gene dosage in synthetic biology, providing precise control over gene expression (17,18), something that is becoming increasingly relevant with the rise of mRNA medicine and gene therapy. The deregulation of miRNA has been implicated in disease such as the neurodegeneration in Parkinson's (9,19) or Alzheimer's disease (10,11,20,21) in *H. sapiens*. Beyond pathological mechanisms, miRNAs are involved in physiological processes such as ageing, in *H. sapiens* (12–14,22–24).

The role miRNAs play in regulating a tissue's transcriptomic signature in turn implies tissue-specific expression. Indeed, this was originally observed in the landmark study by Landgraf et al. (25) and served as inspiration for the first iteration of the miRNATissueAtlas in 2016 (25,26). While it was widely used by the research community, the use of microarray technology limited it to known miRNA and prohibited the exploration of isomiRs. This motivated us to generate the next iteration of the miRNATissueAtlas in 2022 (27) and its specialized isomiRDB (28). Using second generation NGS (Next Generation Sequencing), further, we expanded the collection to more tissues and included *M. musculus*. Using a high-quality, donor matched set of tissues allowed us to explore not only the tissue-specificity of both established and novel miRNA but also nine other classes of non-coding RNA. The inclusion of *M. musculus* allowed us to determine that this tissue-specific distribution is conserved between species, with homologous miRNA having highly correlated tissue-specificity indices. This suggested that miRNA tissue-specificity is evolutionarily conserved and thus critical to the function of tissues.

As a result of their importance to tissue function and disease, the sequencing data collected for non-coding RNA has been growing at an exponential rate. Based on statistics from the NCBI Sequence Read Archive (29), the total number of such publicly available datasets have grown from 27 331 in 2022 to 46 468 in 2024 (SRA, https://www.ncbi.nlm.nih.gov/sra, accessed 27/08/2024). Despite the rapid data growth, our understanding of overall non-coding RNA expression is still far from being complete. As a source to improve that understanding, atlas level resources for miRNA expression data continued to emerge. Notable examples include the human cell-line microRNAome (30), DIANA-miTED (31), isomiRdb (28) and miSRA (https://arn.ugr.es/misra/). Other atlases include sub-sections of the FANTOM project (32), and the Immunological Genome Consortium (33), both notable for their multi-omics approach. So far, however, a label-harmonized resource for different classes of non-coding RNA from *H. sapiens* and *M. musculus* for physiological tissues, cell lines and extracellular vesicles from current publicly available sequencing datasets is lacking.

Our previous repositories with high quality labelling represent a perfect framework to now add the 61593 samples comprising 799 billion reads and annotate them in a uniform manner. Of note, instead of following the previous naming schema we term our update resource as miRNATissueAtlas 2025. Moving away from a classical version numbering by adding the year might help researchers to immediately gauge the recency of the resource they are using. We thus will use this naming convention, containing the year of publication, for all coming databases and web services.

## Material and methods

The central goal of the new release of the miRNATissueAtlas is to have an up-to-date comprehensive collection of data. We thus obtain a list of samples Accessions from the SRA using the search strings: ‘Search (Homo sapiens [Organism]) AND ‘"mirna seq’‘"[Strategy] Filters: Public; single; fastq’ and ‘Search ( (Mus musculus [Organism]) AND ’‘"mirna seq’"[Strategy] AND Filters: Public; single; fastq’ on the 16th of January 2024. Using the list of Accessions, we use pysradb (https://github.com/saketkc/pysradb) to fetch the meta-data available on SRA, and the SRAToolkit (https://github.com/ncbi/sra-tools) to download the fastq files. Adapter trimming is required before reads from fastq files can be mapped to a genome. Since adapter trimming is highly dependent on library preparation method, and since there are multiple such methods for single-end sequencing, we employ findadapt (34). Findadapt is an automated library trimming tool that determines adapter sequences by sampling reads from the fastq file. As a preliminary QC step, we discarded fastq files where findadapt could not detect an adapter sequence. On top of that we perform sequence-based filtering: fastq files that had either <1 million reads, had an exome percentage of >1% or a genome coverage of >1% were discarded.

We use GRCh38 for *H. sapiens* and GRCm39 for *M. musculus* for alignment and subsequent exome and genome coverage percentage calculations. For non-coding RNA quantification we use the miRMaster2 pipeline (35) with the following ncRNA databases: miRBase version 22.1 (36), Ensemble ncRNA version 100 (37), RNACentral version 15 (14), GtRNAdb version 18.1 (13) and NONCODE version 5 (38).

Consistent labelling is critical to making use of ncRNA expression data. Since we found the meta-data on SRA and Biosample to be incomplete, we used geofetch (https://github.com/pepkit/geofetch) to obtain sample information for accessions having a Gene Expression Omnibus entry (39). We combined the text information mined from GEO into a string and used python scripts to map specific keywords from these strings to their Biotype, Organ and Tissue labels. To map keywords to label we manually parsed through the mined text. We ensured that only a single label was used to refer to a single organ/tissue throughout all the datasets, thus harmonizing labels across the entire resource. If we were unable to recover the complete labelling for a sample, we did not include it in the resource. We removed samples with empty values for labels, with the exception of Sex, where we labelled missing values as ‘Unknown’.

Leveraging the labelling, we calculate the tissue specificity index (TSI) corresponding to each of the organs based on the reads per million mapped reads (rpmm) from the samples. We perform TSI calculations separately for the physiological tissues and cell lines as the former is derived from living organism while the latter represent model systems. While we expect the model system to approximate the expression in physiological tissue, we also expect there to be significant differences. The TSI table represents a central result and is available for download for all classes of non-coding RNA from the database website. TSI is calculated according to the following equation:


\begin{equation*}ts{{i}_j} = \mathop \sum \limits_{i = j}^N \frac{{1 - {{{\bar{x}}}_{j,i}}}}{{N - 1}}\end{equation*}

Here *j* corresponds to a ncRNA, *i* corresponds to an organ, *N* corresponds to the total number of organs considered and $\bar{x}_{j,i}$ corresponds to the mean or median expression for the ncRNA *j* for the organ *i*.

Finally, we reimplemented the backbone for handling this data from the ground up. Our previous implementation was written with hundreds of samples in mind, whereas this iteration is approximately 250 times the size. The previous data tables are still available. The raw and normalized data matrices from all the samples in the new database is available in h5ad format (40). For data analysis, we use snakemake 8.16.0, python 3.8.16, matplotlib 3.7.2, matplotlib-venn 1.1.1, scikit-learn 1.3.0, scipy 1.10.1, pandas 2.0.3, plotly 5.6.0, pyarrow 16.1.0, anndata 0.9.2, mieaa 0.3.0, tqdm 4.66.5 and dash_cytoscape 0.2.0. The webserver was set up using the Django Python web framework v3.2.18 and the PostgreSQL database v14.5. It is deployed and managed as a container using docker v23.0.6 and docker-compose v2.29.2. For development of the website, we utilized a django framework 3.2.18, django-bootstrap-v5 1.0.11, JavaScript ES6, datatables 1.11.5, plotly 5.6.0 and jquery 3.5.1.

## Results and discussions

### Expanded data scope with meta-data harmonization

**Figure 1.**
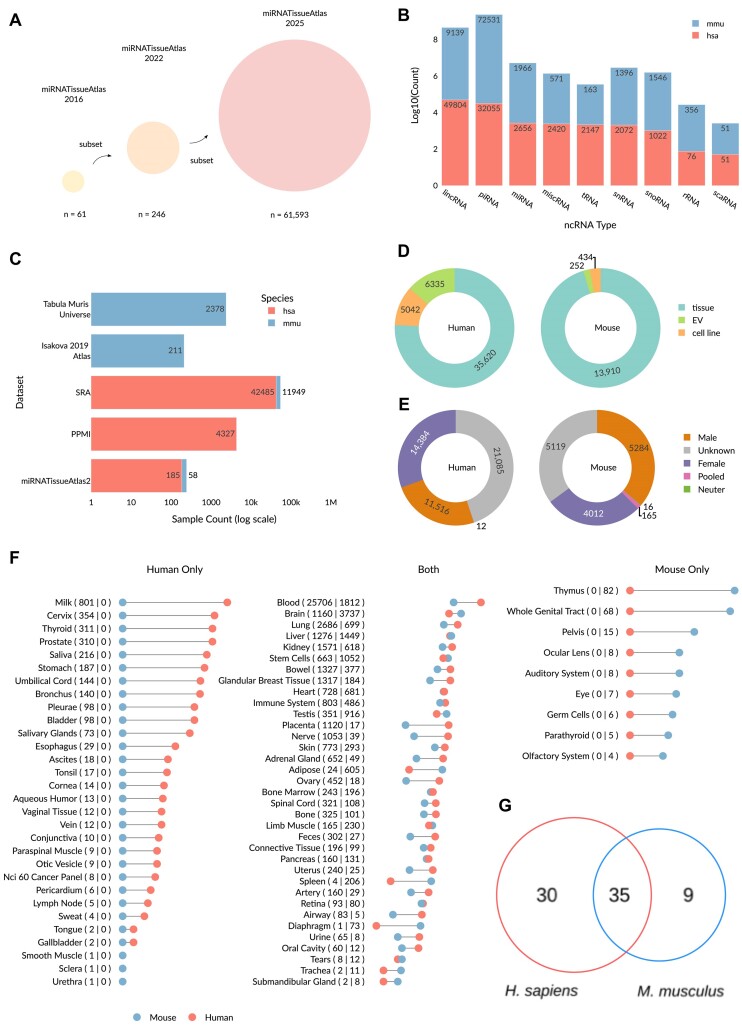
Overall description of the data available. (**A**) Exponential growth in available small-RNA NGS dataset. (**B**) Non-coding RNA type and corresponding number included in dataset from *H. sapiens* (hsa) and *M. musculus* (mmu). (**C**) No. of samples from each dataset included in database for *H. sapiens* (hsa) and *M. musculus* (mmu). (**D**) No. of samples corresponding to tissue, extracellular vesicle and cell line from both *H. sapiens* and *M. musculus*. (**E**) No. of samples corresponding to sex. Samples without sex data were labelled ‘Unknown’. ‘Pooled’ refers to samples where both Male and Female samples were combined. (**F**) Lollipop plot showing the number of samples corresponding to each organ group from *H. sapiens* and *M. musculus*. The columns are split based on if the organ group was found in only human, both human and mouse and only in mouse. For each label on the *y*-axis, the exact counts are mentioned as (*H. sapiens* samples | *M. musculus* samples) next to the organ group. (**G**) Venn diagram of organ common between and exclusive to *H. sapiens* and *M. musculus* sets based on tissue subset from 1d.

While miRNA sequencing data are available for dozens of organisms, we cantered the miRNATissueAtlas to *H. sapiens* and *M. musculus*, likely the best studied organisms in terms of miRNA research. From a starting point of 84 913 samples, of which 65 914 are derived from *H. sapiens* and 18 999 from *M. musculus*, our stringent QC filtering retained the 72.5% highest quality samples as the basis for miRNATissueAtlas 2025. With 46 997 samples from *H. sapiens* and 14 596 samples for *M. musculus*, totalling 61 593 samples, this likely represents one of the largest sets of label-harmonized, pre-processed samples derived from *H. sapiens* and *M. musculus*. The quantity of the data is given by a total of 799 billion reads that we uniformly processed. Given the size and breadth of our dataset, evolutionary variation is expected to be present in the dataset, both between and within species. This variation is encapsulated partially in the expression of isomiRs (9). We make the matrices of abundant isomiRs (>1 rpmm in at least 1% of samples) with a uniform nomenclature available to users, with 123 938 and 100 253 isomiRs for *H. sapiens* and *M. musculus* respectively. Although the title of our resource, miRNATissueAtlas, implies that we consider only miRNAs, we also detect and report other classes of non-coding RNA and therefore include piRNA, miscRNA, tRNA, snRNA, snoRNA, rRNA, scaRNA and lincRNA expression matrices as well (Figure 1B). For historical reasons, we decided to stay with the original name, also avoiding confusion among researchers who have been using the previous iterations of our resource. Apart from that, among all non-coding RNA, the best-studied miRNAs remain the focus of our database.

While most of the samples are derived from SRA for both *H. sapiens* and *M. musculus* (42485 and 11949 respectively), we want to highlight several large-scale data sets we contributed. For example, the Parkinson's Progression Marker Initiative cohort (19) represents 16.8% of all *H. sapiens* samples derived from Blood (4327 out of 25 706). For *M. musculus*, we added the Isakova 2019 mouse tissue atlas, a study demonstrating the ability for ncRNA to classify tissues in *M. musculus*, thus highlighting their tissue specific nature (41). We have also contributed significantly to aging datasets with studies related to the Tabula Muris RNA sequencing studies (24,42–44) (n = 2378). Taken together, our collaborators and us generated 2647 out of 14 596, i.e. 18.1% of all *M. musculus* miRNA datasets sequenced so far (Figure 1C).

The contrast between number *H. sapiens* samples compared to *M. musculus* samples is stark, with an approximately 3-fold difference. We recovered definite Sex labels for 55.1% samples in *H. sapiens* and 64.9% samples in *M. musculus*. Interestingly, we see similar proportions of labelling for Male and Female in both species (24.5% male, 30.6% female in *H. sapiens* and 36.2% male, 27.4% female in *M. musculus*) (Figure 1E). However, differences in sample count between species are maintained when we decompose the datasets by Biotype; most cell lines are derived from *H. sapiens* (5042 in *H. sapiens* versus 434 in *M. musculus*). This is reasonable as *M. musculus* is itself meant to be a model organism for *H. sapiens;* creating models of a model organism is somewhat redundant. The label-harmonization of organs and tissues enable us to compare similarities between *H. sapiens* and *M. musculus*: out of 74 total organs, 35 are common to both species with 30 being specific to *H. sapiens* and only 9 being specific to *M. musculus* (Figure 1G).

Zooming into finer tissue labels, which are sublocations and cell lines assigned to organ labels, we see the degree of similarity flip: only 59 labels match between *H. sapiens* and *M. musculus* with 165 and 149 labels being unique to each species respectively (Supplementary Figure S1A). Examination of the frequencies of each individual label (Supplementary Figure S1B,C), we see that 103 of the 165 *H. sapiens* specific labels are coming from cell lines. Meanwhile, 144 out of 149 of all *M. musculus* specific labels are derived from the tissue subset.

This pattern reveals in part the motivation behind sampling certain tissues; we want to sample cell lines to approximate *in vivo* tissue and therefore it is reasonable to sample cell lines. However, *M. musculus* tissue is already a model for *in vivo H. sapiens* tissue, and therefore, it is not reasonable to sample cell lines, which would be a redundant model of a model organism. The pattern also reveals the logistics behind sampling, e.g. it is much more difficult to sample *H. sapiens* embryonic development because of ethical concerns than it is to sample *M. musculus*. This general pattern shows itself in the harmonized organ sets as well. Approximately 54.7% of all *H. sapiens* sample is derived from blood, while it only represents 12.4% of all *M. musculus* samples.

The harmonized set of organs and tissues opens avenues of cross-species investigation based on current data. The mismatches, on the other hand, highlights blind spots where data can be collected to form a clearer picture of the role of miRNA in those tissues. The development of embryos, for example, is one such critical area of research. Of note, there appears to be a dearth of non-coding data derived from organoids, which are quickly becoming an important model system, apart from *M. musculus* and cell lines.

### TSI distributions across tissue and cell lines

**Figure 2.**
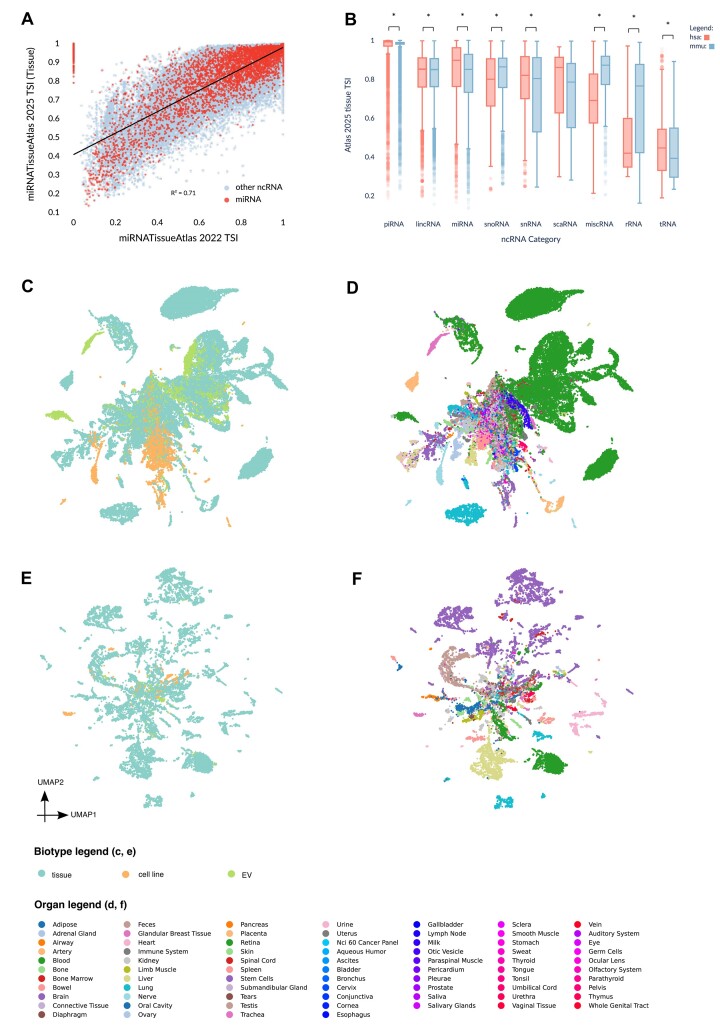
TSI and expression summary of non-coding RNA for tissue subset. (**A**) Comparison between TSI calculated for miRNATissueAtlas 2025 versus the previous iteration of the miRNATissueAtlas 2022. R^2^ value is reported from fitting a simple linear regression model. (**B**) TSI distribution for all included non-coding RNA groups for the tissue subset of miRNATissueAtlas 2025 for both *H. sapiens* (hsa) and *M. musculus* (mmu). (**C**) UMAP (Uniform Manifold Approximation and Projection) overview of miRNATissueAtlas 2025 (2025) *H. sapiens* dataset using tissue-specific miRNA subset, colored by biotype group. (**D**) UMAP overview of miRNATissueAtlas 2025 (2025) *H. sapiens* dataset using tissue-specific miRNA set, colored by organ group. (**E**) UMAP overview of miRNATissueAtlas 2025 *M. musculus* dataset using tissue-specific miRNA set, colored by biotype group. (**F**) UMAP overview of miRNATissueAtlas 2025 *M. musculus* dataset using tissue-specific miRNA set, colored by organ group.

As previously mentioned, non-coding RNA tend to be expressed in a tissue specific manner, a property likely derived from their role as regulatory molecules. Tissue-specific expression, therefore, is taken to be a signal for functionality, marking non-coding RNA specific to certain tissues as important to the functional identity of those tissues. Our previous iteration of the miRNATissueAtlas (2022) calculated TSI based on expression in high-quality and source-matched matched tissues. We build upon this foundation by calculating the TSI from our expanded set in the miRNATissueAtlas 2025. Interestingly, plotting the TSI from the expanded miRNATissueAtlas 2025 and the previous release miRNATissueAtlas2 from 2022, we identify a striking correlation, with an R^2^ value of 0.71 (Figure 2A). suggesting that the tissue-specific expression patterns are conserved in the expanded set of tissues. Comparing the TSI distribution of different ncRNA classes between species, we note similarities between the median TSI values of *H. sapiens* and *M. musculus* for piRNA, lincRNA, miRNA, snoRNA, snRNA, scaRNA and tRNA. However, statistical testing shows that the distributions are significantly different (except for scaRNA, Supplementary Table S1), highlighting that TSI is only partially conserved between *H. sapiens* and *M. musculus*. Interestingly, piRNA appear to have the highest median for TSI compared to other classes of non-coding RNA. This is likely explained by their specific expression in the testis (27). Conversely, tRNA has the lowest median for TSI, which is explained by its universal role in protein translation in all tissue. We also find there to be a high correlation of TSI calculated from tissues with those calculated from cell lines, with an R^2^ value of 0.66 (Supplementary Figure S2A), suggesting that the miRNA function is at least partially conserved between tissue and cell lines.

We select the set of miRNAs that have a TSI value over 0.8 and which have been detected over a threshold of 10 rpmm in at least 75% of the samples in an organ group to perform a PCA embedding. Using the first 20 principal components, we calculate a UMAP embedding to give an overview of the relationship of organs and biotype to each other (Figure 2C–F). As expected, blood forms the most dramatic cluster in *H. sapiens*. We notice that cell lines form their own clusters, while EV is primarily overlapping with samples derived from blood. Interestingly, cell lines clustering away from tissues was previously observed in (45). This contrasts with the correlation of TSI between physiological tissues and cell lines and suggests that while the miRNA themselves are expressed in a specific manner, the overall expression is not completely preserved between cell lines and physiological tissues, especially since many cell lines are immortalized cells derived from tumors. Meanwhile, the closeness of EV to Blood is likely due to EVs primarily being derived from blood in *H. sapiens*. Overall, however, this suggests that the expression patterns are still more similar within the biogroups than between. We are similarly able to separate the organs in *M. musculus* samples, where instead of blood, brain forms the most visibly separated cluster. However, it is notable that the data is not perfectly separable from the UMAP even after choosing the most tissue specific miRNA, likely due to the unbalanced sets in the data and the need for future higher resolution meta-data extraction. To maximize the usability of this dataset by interested researchers, we make the data available in h5ad format on the website, split by the class of non-coding RNA, with the meta-data included in the h5ad files.

### Use case: mouse-human cross-organ similarity

To demonstrate the capacities of the miRNATissueAtalas 2025, we performed a use-case describing the cross-organ similarity between mice and humans. In preclinical studies, *M. musculus* serves as an important model organism for *H. sapiens*. Unfortunately, the number of therapies that make it all the way through the development pipeline from preclinical studies to the market is low and has been declining over time, with the failure rates happening at the end stages of Phase II and Phase III (the most expensive stages), suggesting that too many false positives are let through in the earlier, less expensive phases (46,47). To reduce the number of false positives in the early stages of research and development of miRNA therapeutics development, a cross-species comparison of miRNA expression might be useful to identify the miRNA patterns might translate and which might not.

**Figure 3.**
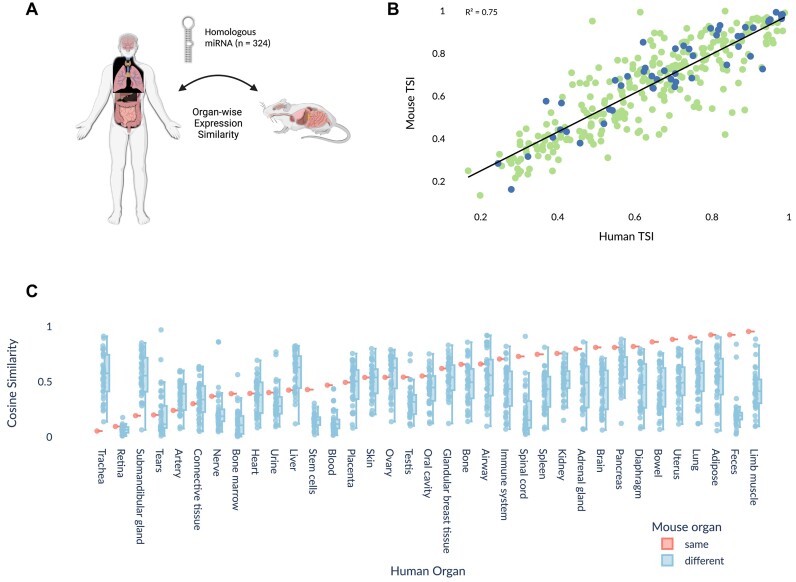
Example use case of cross-species analysis using miRNA set of miRNATissueAtlas 2025. (**A**) Select homologous miRNAs based on calculations made in the TargetScan database. Using this set, we can compare the expression pattern per organ between *H. sapiens* and *M. musculus*. (Created with BioRender.com) (**B**) Correlation between TSI between *H. sapiens* and *M. musculus* for the set of homologous miRNAs. The miRNAs where the max median expression of organ match between human and mouse are highlighted on top of other homologous miRNAs. (**C**) Pairwise cosine similarity distribution of homologous miRNA for harmonized set of 35 organs. Each dot represents a pairwise comparison between a *H. sapiens* and *M. musculus* organ. The single dot on the left of each group on the x-axis is the comparison where organs are identical between *H. sapiens* and *M. musculus*. E.g. for the dots corresponding to blood on the *x*-axis the dot on the left represent the comparison with blood for *M. musculus* while dots on the right correspond to comparison between blood and brain, kidney, liver etc. correspondingly.

So, a potential use case is to compute and to list miRNA expression patterns that are conserved between the species (and, therefore, likely to translate between species). To calculate this, we obtain the miRNA names from TargetScan families (4). We then identify miRNA that have unified naming belonging the same family and obtain 324 miRNAs. Plotting the TSI value of matched miRNA found in *H. sapiens* and *M. musculus* (Figure 3B), we find that they are highly correlated, with an R^2^ value of 0.75. When we look at the maximum median expression of each of these miRNAs and compare the organ of maximum median expression between *H. sapiens* and *M. musculus* we see that 52 out of 324 miRNAs having matching organ. It is also of note that the miRNAs that are matching by organ appear to be clustered to the top right of the scatterplot (Figure 3B), meaning that homologs that have higher TSI in both *H. sapiens* and *M. musculus* also tend to have matching tissues.

To create an overview of similarity, we do pairwise comparisons of expression patterns in the organs. The chosen metric here is cosine similarity as it summarizes how similar the direction is of a set of expression values without being dependant on their magnitude. In Figure 3C, we highlight how the cosine similarity of matching organs between *H. sapiens* and *M. musculus* (red) compared to the other organs (blue). We see, for example, that limb muscle has the highest similarity between the two organisms, followed by feces. While the latter is surprising, it aligns with the results that the microbiome of *H. sapiens* and *M. musculus* is highly similar at the phylum level (48). While many factors account for the diversity of the microbiome, we increasingly find that miRNA can influence the gut microbiota composition (49). Taken together, it is probable that the set of miRNAs homologous between *H. sapiens* and *M. musculus* is a contributing factor. However, a deeper analysis is beyond the scope of this paper.

### Limitations

There are two limitations to keep in mind when utilizing this atlas. The first is that, while the tissue and organ meta-data labels were harmonized, they are by no means a complete description of the conditions under which the experiments where conducted. This atlas represents the entire range of expression data on all miRNA sequencing experiments that are publicly available. That includes conditions such as disease, knock-out, overexpression experiments, different ages, sexes etc. The effects of organ identity generally overwhelm these effects in bulk data. However, they introduce heterogeneity that must be kept in mind when using the data. The second is the unbalanced nature of the labels. As previously mentioned, the logistics and practicality of conducting experiments significantly impacts the origin of the samples. This property of the dataset might skew the results of analyses if not considered. Last not least, experimental bias as the use of different sequencing protocols might have a significant impact on the results. Another type of bias is the limitation to *M. musculus* and *H. sapiens*. With growing data sets becoming available for other species, we are confident to get more insights into the miRNA tissue specificity and whether it is evolutionary conserved between those other species as well.

## Permissions

Graphical abstract, Figure 3A Biorender Publication and Licensing Rights agreement numbers: TZ27ERK5LK and KB279LDLQW respectively.

## Supplementary Material

gkae1036_Supplemental_File

## Data Availability

All data are available through the miRNATissueAtlas 2025 web repository at https://www.ccb.uni-saarland.de/tissueatlas2025.
