# Supplementary material for: miRNATissueAtlas 2025: an update to the uniformly processed and annotated human and mouse non-coding RNA tissue atlas
Source: Nucleic Acids Res. 2024 Nov 14;53(D1):D129–37. doi: 10.1093/nar/gkae1036 (PMC11701691; doi:10.1093/nar/gkae1036)
Supplement: gkae1036_Supplemental_File [file gkae1036_supplemental_file.pdf]

Supplementary Figure 1a

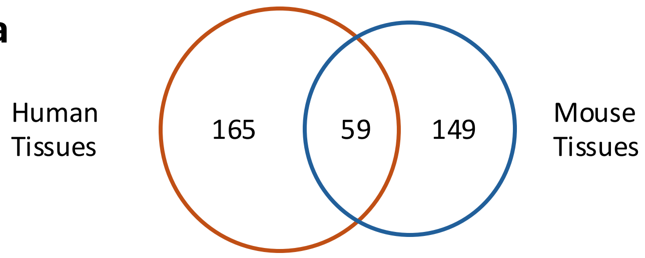

Supplementary Figure 1b

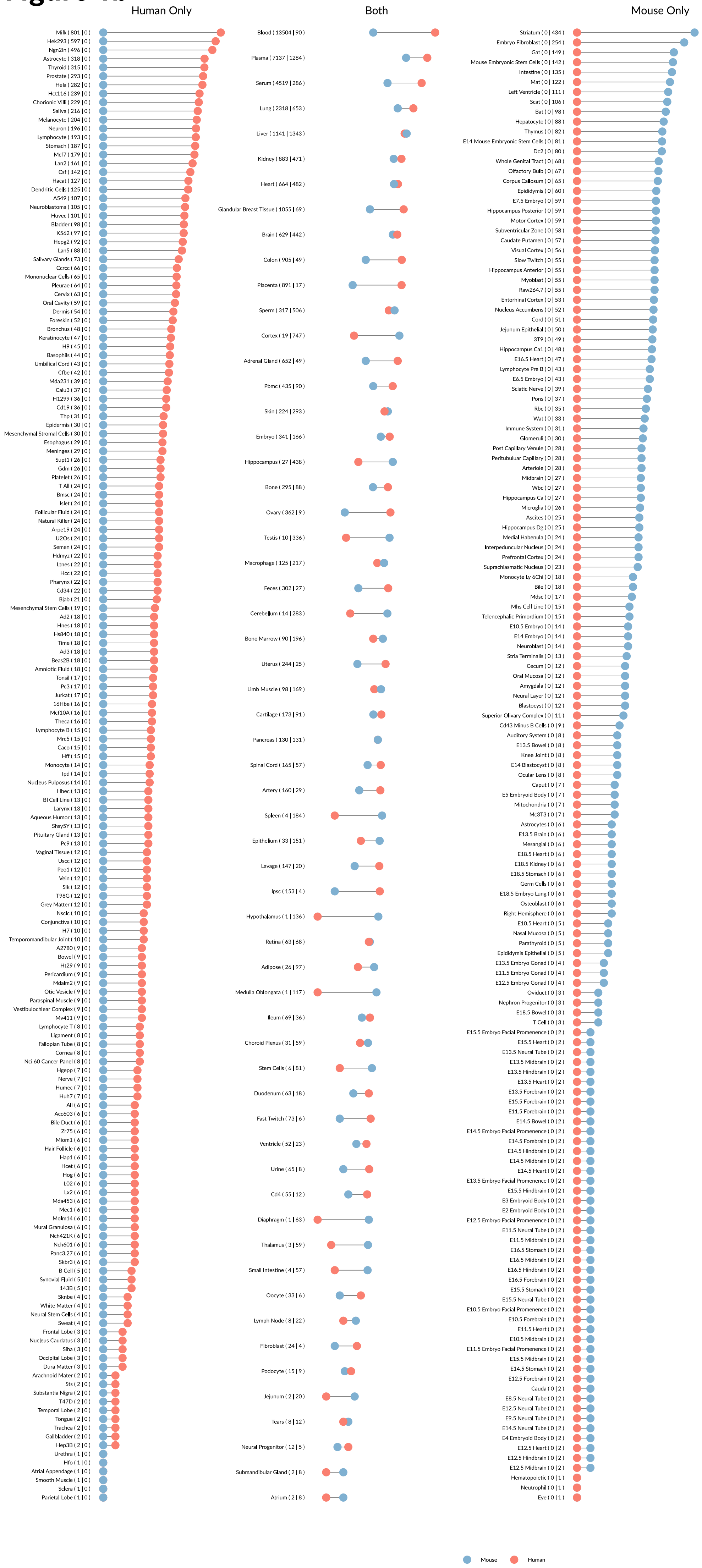

Supplementary Figure 1c

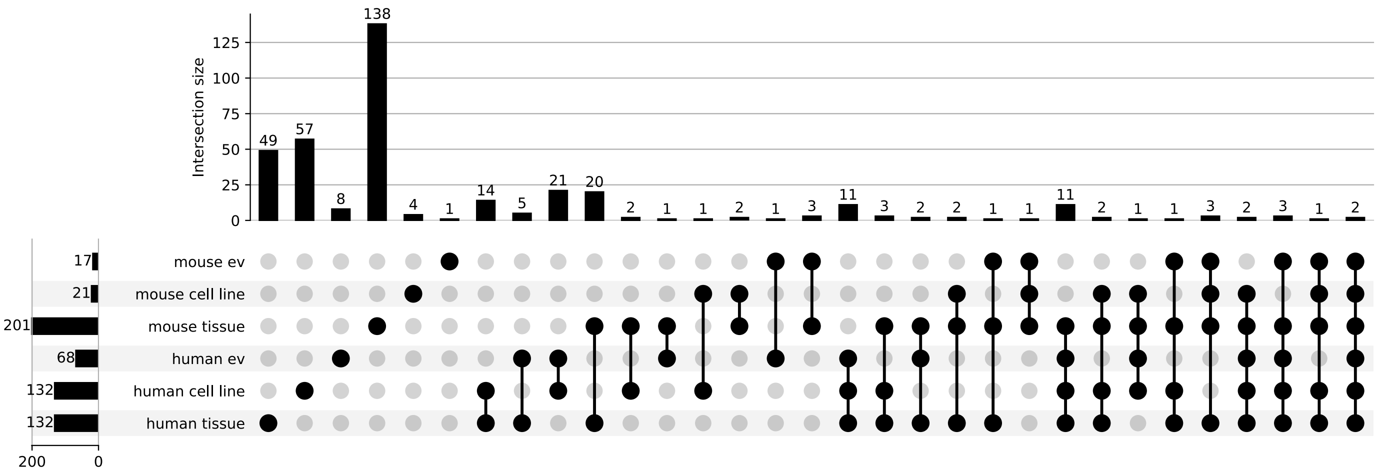

Supplementary Figure 2a

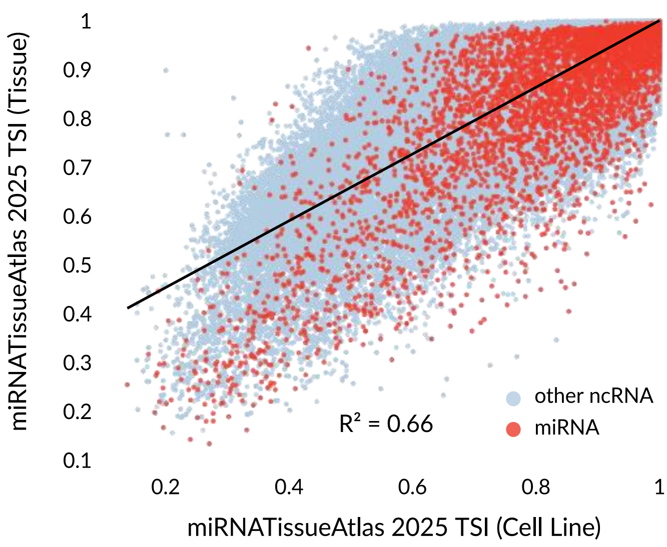

Supplementary Figure Captions

Supplementary Figure 1: (a) Venn diagram showing overlap of tissue labels between *H. sapiens* and *M. musculus.* (b) Number of tissue labels deriving from each biotype that is found in only *H. sapiens, M. musculus* or shared across both species. (c) Upset plot of tissue labels derived from tissue, cell lines and extracellular vesicles in *H. sapiens* and *M. musculus.*

Supplementary Figure 2: Correlation between tissue-specificity index calculated for physiological tissues and cell lines from miRNATissueAtlas 2025 for all non-coding RNA for *H. sapiens* and *M. musculus*. Red dots highlight miRNA. The line represents a simple linear regression fit with an R^2 value of 0.66.

Supplementary Table 1

ncRNA type	Mann Whitney U statistic for hsa vs. mmu TSI	P-value	No. of hsa ncRNA	No. Of mmu ncRNA	hsa TSI median	mmu TSI median	hs TSI IQR	mmu TSI IQR
miRNA	3019169.5	8.719E-20	2656	1966	0.89725	0.8506	0.1993	0.1957
lincRNA	233684743.0	4.442E-05	49804	9139	0.8516	0.8493	0.1502	0.1447
miscRNA	378628.0	1.627E-63	2420	571	0.69025	0.8723	0.2515	0.1452
piRNA	1122986413.0	1.668E-18	32055	72531	0.9825	0.985	0.1205	0.0094
rRNA	8984.0	4.233E-06	76	356	0.41935	0.76545	0.2479	0.4532
scaRNA	1546.0	1.011E-01	51	51	0.8605	0.7853	0.2787	0.3107
snoRNA	683932.5	8.048E-09	1022	1546	0.7992	0.86365	0.2438	0.1507
snRNA	1614150.0	6.394E-09	2072	1396	0.81925	0.80365	0.2189	0.3819
tRNA	194306.0	1.857E-02	2147	163	0.4463	0.3928	0.2116	0.2524

Supplementary Table Captions

Supplementary Table 1: Mann Whitney U test comparison forTissue Specificity index distributions between *H. sapiens* (hsa) and *M. musculus* (mmu).
